# Supplementary material for: Lysophosphatidylserine induces necrosis in pressure overloaded male mouse hearts via G protein coupled receptor 34
Source: Nat Commun. 2023 Jul 31;14:4494. doi: 10.1038/s41467-023-40201-4 (PMC10390482; doi:10.1038/s41467-023-40201-4)
Supplement: Supplementary file 1 — Supplementary information [file 41467_2023_40201_MOESM1_ESM.pdf]

Supplementary information for the manuscript entitled:

Lysophosphatidylserine induces necrosis in pressure overloaded male mouse hearts via G protein coupled receptor 34

Ryuta Sugihara et al.

- Supplementary figures (1-8)
- Supplementary Tables (1-4)
- Minimal checklist of lipidomics analysis
- Uncropped scans of blots in supplementary figures (supplementary figure 1, 2 and 7)

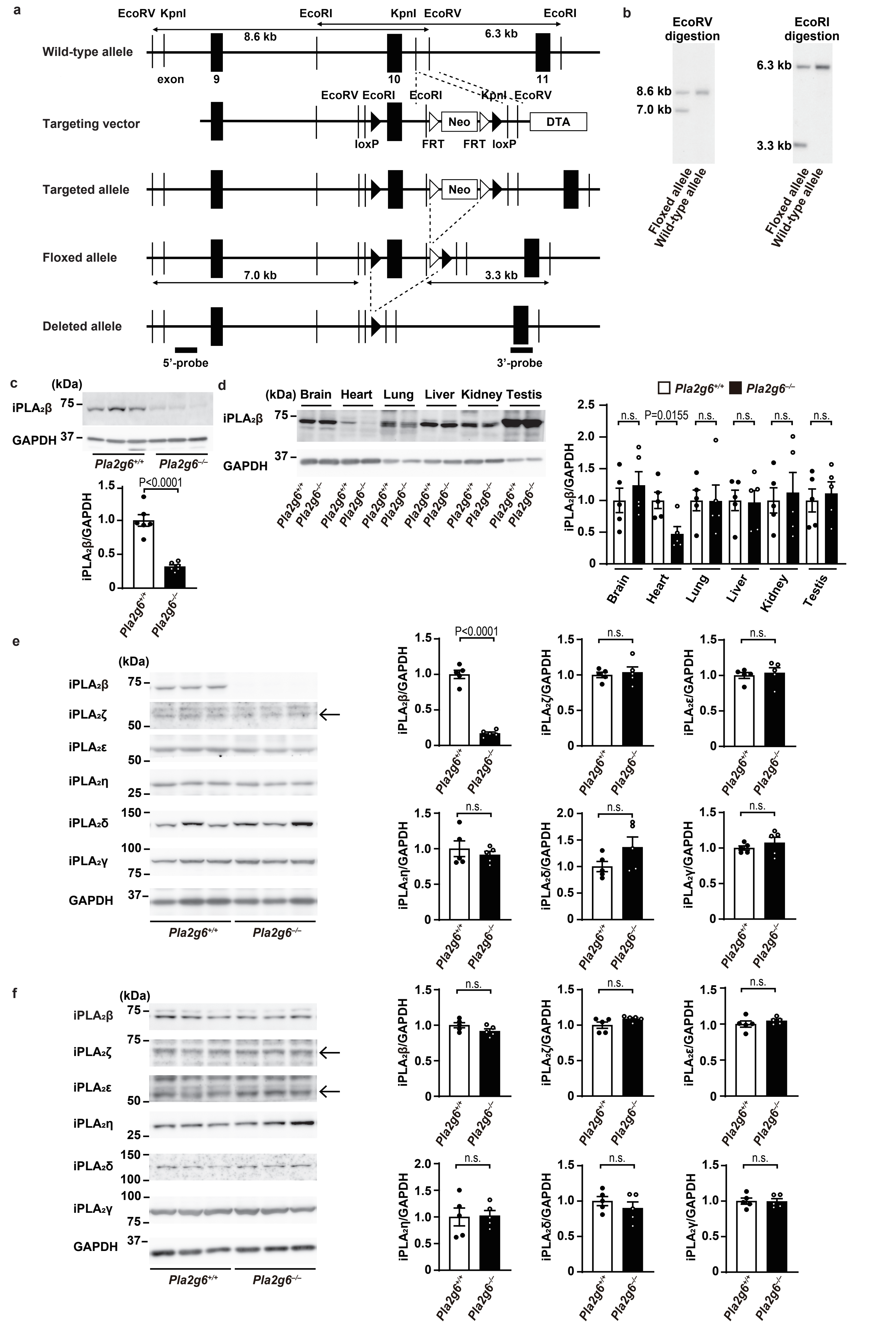

Supplementary Fig. 1: Generation of cardiomyocyte-specific iPLA$_2$β-deficient mice.

a Targeted modification of the *Pla2g6* gene. Schematic structures of the wild-type genomic *Pla2g6* allele, the targeting vector, the targeted allele, the floxed allele after flippase recognition target site (*FRT*)-mediated deletion of neomycin-resistance gene (*Neo*), and the deleted allele after *Cre*-mediated recombination are indicated from top to bottom. The black and white arrowheads indicate *loxP* and *FRT* sites, respectively. The neomycin resistance gene (*PGK-Neo cassette*) was inserted between exon ten and the downstream *loxP* site. The diphtheria toxin A (*DTA*) gene was positioned at the 3' end of the targeting vector for negative selection. The bar labelled 'probe' corresponds to the sequence used for southern blotting. **b** Genomic analysis of embryonic stem (ES) cells. To identify homologous recombinants, genomic DNA extracted from ES cells was digested with EcoRV or EcoRI, and analyzed by southern blotting with the 5' or 3' probe, respectively. Representative images show floxed and wild-type alleles using the 5' probe after EcoRV digestion (left) or using the 3' probe after EcoRI digestion (right). The data are representative of at least nine biologically independent samples. The ES cells with the *Pla2g6*-floxed allele were injected into blastocysts to obtain floxed *Pla2g6* mice. **c** Protein expression levels of iPLA$_2$β in *Pla2g6*$^{+/+}$ and *Pla2g6*$^{-/-}$ cardiomyocytes. Isolated adult cardiomyocyte lysates from *Pla2g6*$^{+/+}$ and *Pla2g6*$^{-/-}$ mice were analyzed by western blotting with anti-iPLA$_2$β antibody. Densitometric analysis is shown in the bottom panel (n = 6, biologically independent samples, P=0.00002). The average value of iPLA$_2$β/GAPDH in *Pla2g6*$^{+/+}$ was set equal to 1.The samples derived from the same experiment and blots were processed in parallel. **d** Protein expression levels of iPLA$_2$β in multiple tissues. Tissue homogenates from *Pla2g6*$^{+/+}$ and *Pla2g6*$^{-/-}$ mice were analyzed by western blotting with anti-iPLA$_2$β antibody. Densitometric analysis is shown in the right panel (n = 5, biologically independent samples). The average value of iPLA$_2$β/GAPDH in each tissue in *Pla2g6*$^{+/+}$ was set equal to 1. The samples derived from the same experiment and blots were processed in parallel. **e** Protein expression levels of iPLA$_2$ subtypes in *Pla2g6*$^{+/+}$ and *Pla2g6*$^{-/-}$ mouse cardiomyocytes. Isolated adult cardiomyocyte lysates from *Pla2g6*$^{+/+}$ and *Pla2g6*$^{-/-}$ mice were analyzed by western blotting using the indicated antibodies. Densitometric analysis is shown in the right panel (n = 5, biologically independent samples, P=0.0000009 in iPLA$_2$ β/GAPDH). GAPDH was used as sample processing controls. The samples derived from the same experiment and blots were processed in parallel. **f** Protein expression levels of iPLA$_2$ subtypes in *Pla2g6*$^{+/+}$ and *Pla2g6*$^{-/-}$ non-cardiomyocytes. Isolated adult non-cardiomyocyte lysates from *Pla2g6*$^{+/+}$ and *Pla2g6*$^{-/-}$ mice were analyzed by western blotting using the indicated antibodies. Densitometric analysis is shown in the right panel (n = 5, biologically independent samples). GAPDH was used as sample processing controls. The samples derived from the same experiment and blots were processed in parallel. The values are presented as the mean ± SEM. The average value of the indicated iPLA$_2$ subtypes in *Pla2g6*$^{+/+}$ was set equal to 1. The data were evaluated using two-sided unpaired Student's t-test. Source data are provided as a Source Data file.

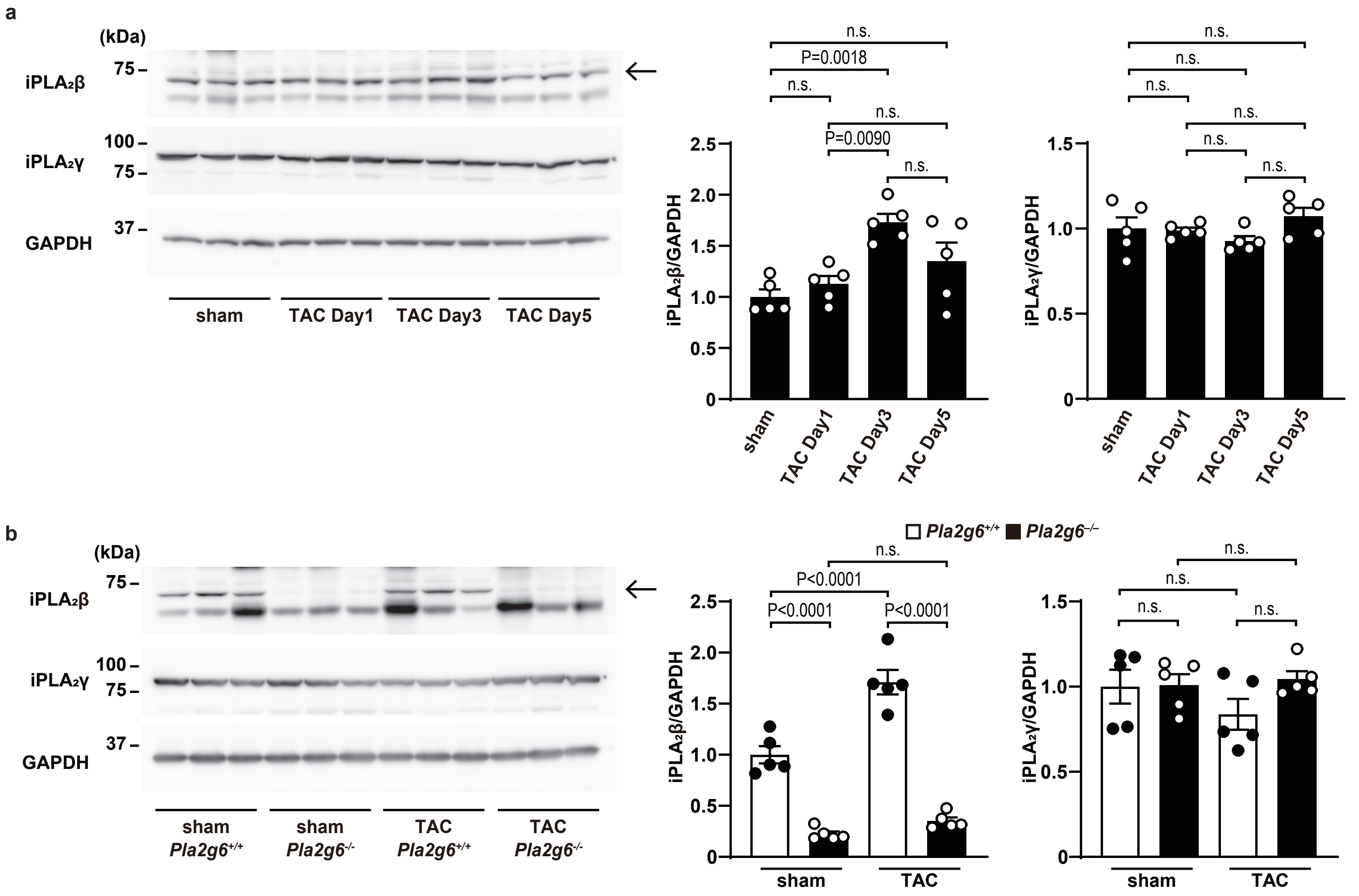

Supplementary Fig. 2: Protein expression level of iPLA$_2$β and iPLA$_2$γ after TAC operation.

a Time course of protein expression levels of iPLA$_2$β and iPLA$_2$γ after TAC operation in wild-type C57BL/6J mouse hearts. **b** Protein expression levels of iPLA$_2$β and iPLA$_2$γ 3 days after TAC operation in *Pla2g6*$^{+/+}$ and *Pla2g6*$^{–/–}$ hearts. *n* = 5, biologically independent samples for each experiment. Densitometric analysis is shown in the right panels. Open and closed bars indicate *Pla2g6*$^{+/+}$ and *Pla2g6*$^{–/–}$ mice, respectively (**b**). The values are presented as the mean ± SEM. The average value of the indicated iPLA$_2$ subtypes in sham group or sham-operated *Pla2g6*$^{+/+}$ group was set equal to 1. The data were evaluated by one-way ANOVA, followed by Tukey-Kramer's *post-hoc* test (Sham *Pla2g6*$^{+/+}$ versus sham *Pla2g6*$^{–/–}$ *P*=0.00001, sham *Pla2g6*$^{+/+}$ versus TAC *Pla2g6*$^{+/+}$ *P*=0.00004, TAC *Pla2g6*$^{+/+}$ versus TAC *Pla2g6*$^{–/–}$ *P*=0.000000006 in iPLA$_2$β/GAPDH, **b**). GAPDH was used as sample processing controls. The samples derived from the same experiment and blots were processed in parallel. Source data are provided as a Source Data file.

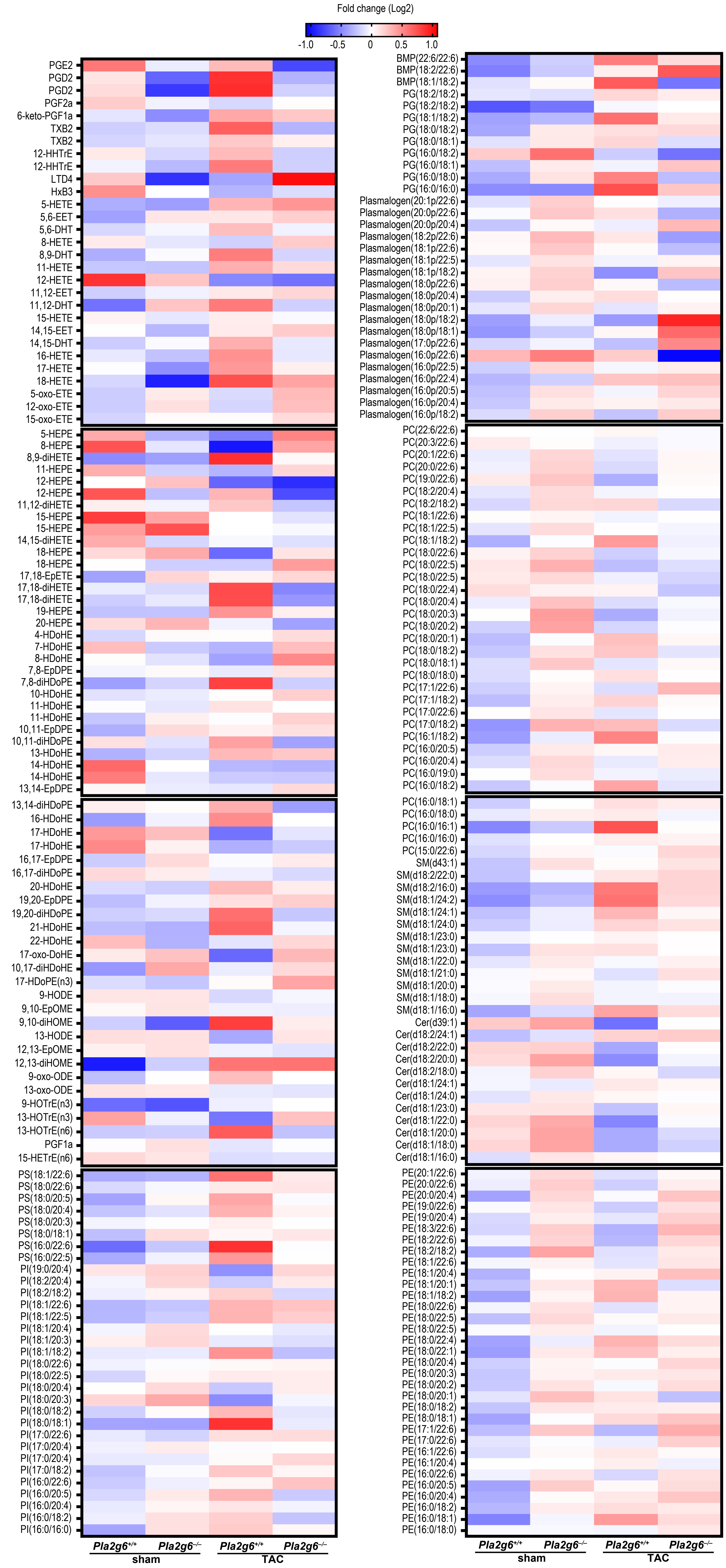

Supplementary Fig. 3: Untargeted and targeted lipidomic analysis in TAC-operated *Pla2g6*$^{-/-}$ heart tissue.

Heat map showing the mean contents of molecular species including arachidonic acid metabolites in the hearts of mice after TAC operation (n = 5, biologically independent samples). Correlation of peak values and color are indicated at the top; High, red and low, blue. PGE, prostaglandin E; PGD, prostaglandin D; PGF, prostaglandin F; TXB, thromboxane B; HHTrE, hydroxyheptadecatrienoic acid; LTD, leukotriene D; HxB, hepoxilin B; HETE, hydroxyeicosatetraenoic acid; EET, epoxyeicosatrienoic acid; DHT, dihydrotestosterone; oxo-ETE, oxo-eicosatetraenoic acid; HEPE, hydroxy/hydroperoxyeicosapentaenoic acid; diHETE, dihydroxyeicosatetraenoic acid; EpETE, epoxyeicosatetraenoic acid; HDoHE, hydroxydocosahexaenoic acid; EpDPE, epoxydocosapentaenoic acid; diHDoPE, dihydroxydocosapentaenoic acid; oxo-DoHE, oxo-docosahexaenoic acid; diHDoHE, dihydroxydocosahexaenoic acid; HDoPE, hydroxydocosapentaenoic acid; HODE, hydroxyoctadecadienoic acid; EpOME, epoxyoctadecenoic acid; diHOME, dihydroxyoctadecenoic acid; oxo-ODE, oxo-octadecadienoic acid; HOTrE, hydroxyoctadecatrienoic acid; HETrE, hydroxyeicosatrienoic acid; PS, phosphatidylserine; PI, phosphatidylinositol; BMP, bis(monoacylglycero)-phosphate; PG, phosphatidylglycerol; PC, phosphatidylcholine; SM, sphingomyelin; Cer, ceramide; PE, phosphatidylethanolamine.

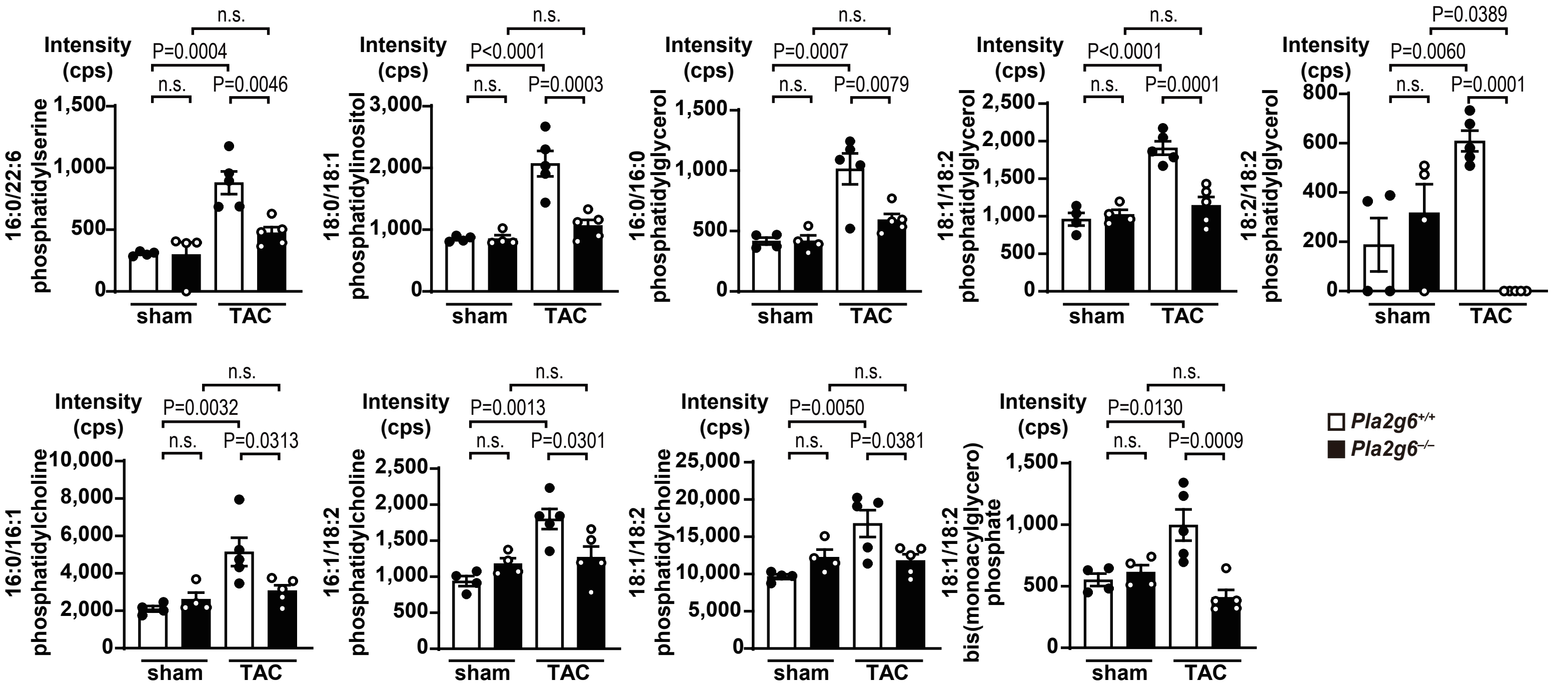

Supplementary Fig. 4: Quantity of lipid mediator candidates in mouse hearts after TAC operation.

Quantity of lipid mediator candidates in mouse hearts after TAC operation. *n* = 4 (sham-operated *Pla2g6*$^{+/+}$), 4 (sham-operated *Pla2g6*$^{–/–}$), 5 (TAC-operated *Pla2g6*$^{+/+}$), and 5 (TAC-operated *Pla2g6*$^{–/–}$). Open and closed bars indicate *Pla2g6*$^{+/+}$ and *Pla2g6*$^{–/–}$, respectively. Data are expressed as the mean ± SEM. The data were evaluated by one-way ANOVA, followed by Tukey-Kramer's *post-hoc* test (Sham *Pla2g6*$^{+/+}$ versus TAC *Pla2g6*$^{+/+}$ *P*=0.00006 and *P*=0.00003 in 18:0/18:1 phosphatidylinositol and 18:1/18:2 phosphatidylglycerol, respectively). Source data are provided as a Source Data file.

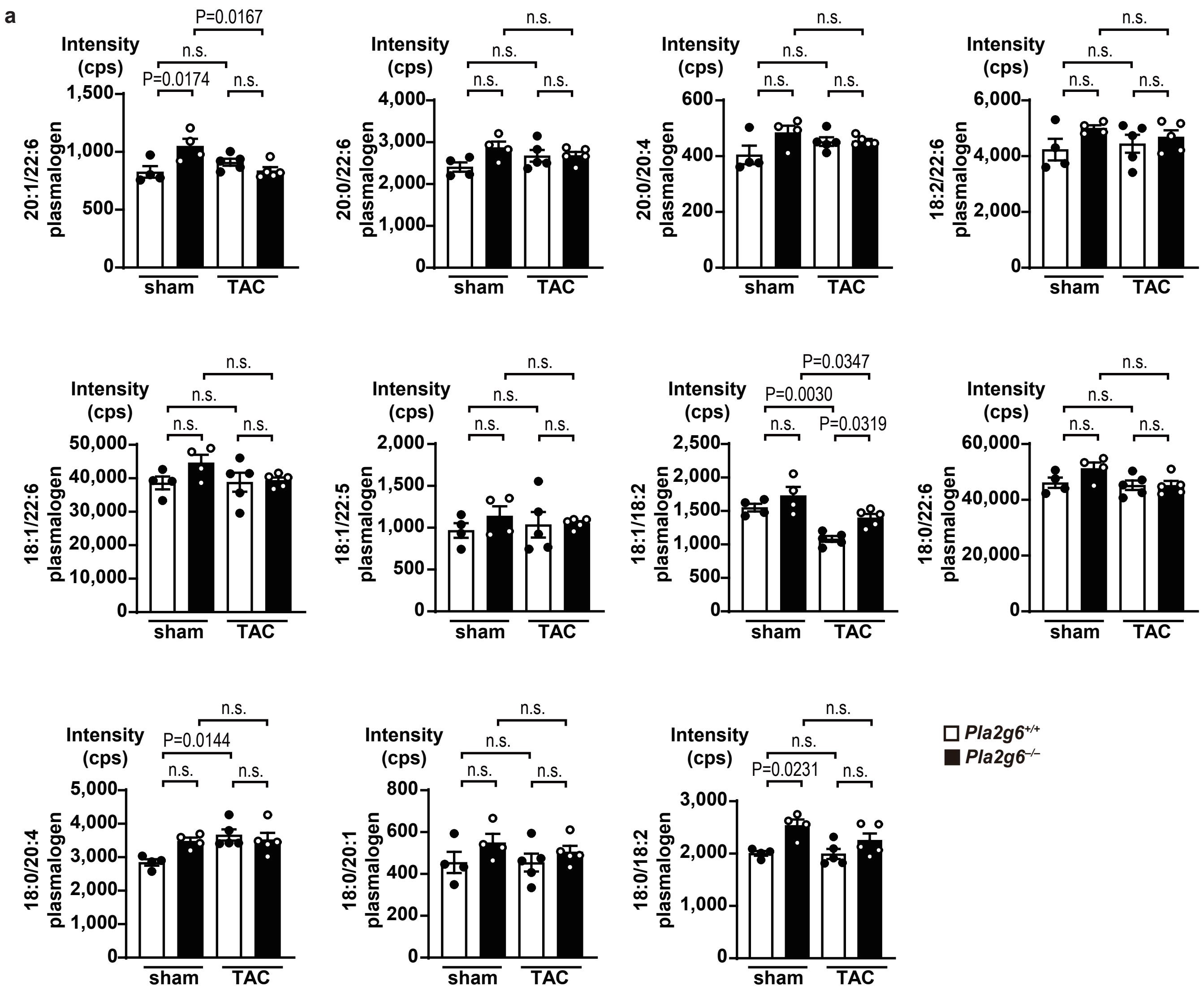

Supplementary Fig. 5: Quantity of plasmalogens, 18:1 and 18:2 free fatty acid in mouse hearts after TAC operation.

a Quantity of plasmalogens detected in mouse hearts after TAC operation. **b** Quantity of 18:1 and 18:2 free fatty acids, which are products derived from 18:1/18:2 plasmalogen. n = 4 (sham-operated *Pla2g6*$^{+/+}$), 4 (sham-operated *Pla2g6*$^{-/-}$), 5 (TAC-operated *Pla2g6*$^{+/+}$), and 5 (TAC-operated *Pla2g6*$^{-/-}$), biologically independent samples. Open and closed bars indicate *Pla2g6*$^{+/+}$ and *Pla2g6*$^{-/-}$, respectively. Data are expressed as the mean ± SEM. The data were evaluated by one-way ANOVA, followed by Tukey-Kramer's *post-hoc* test. Source data are provided as a Source Data file.

a (Continued)

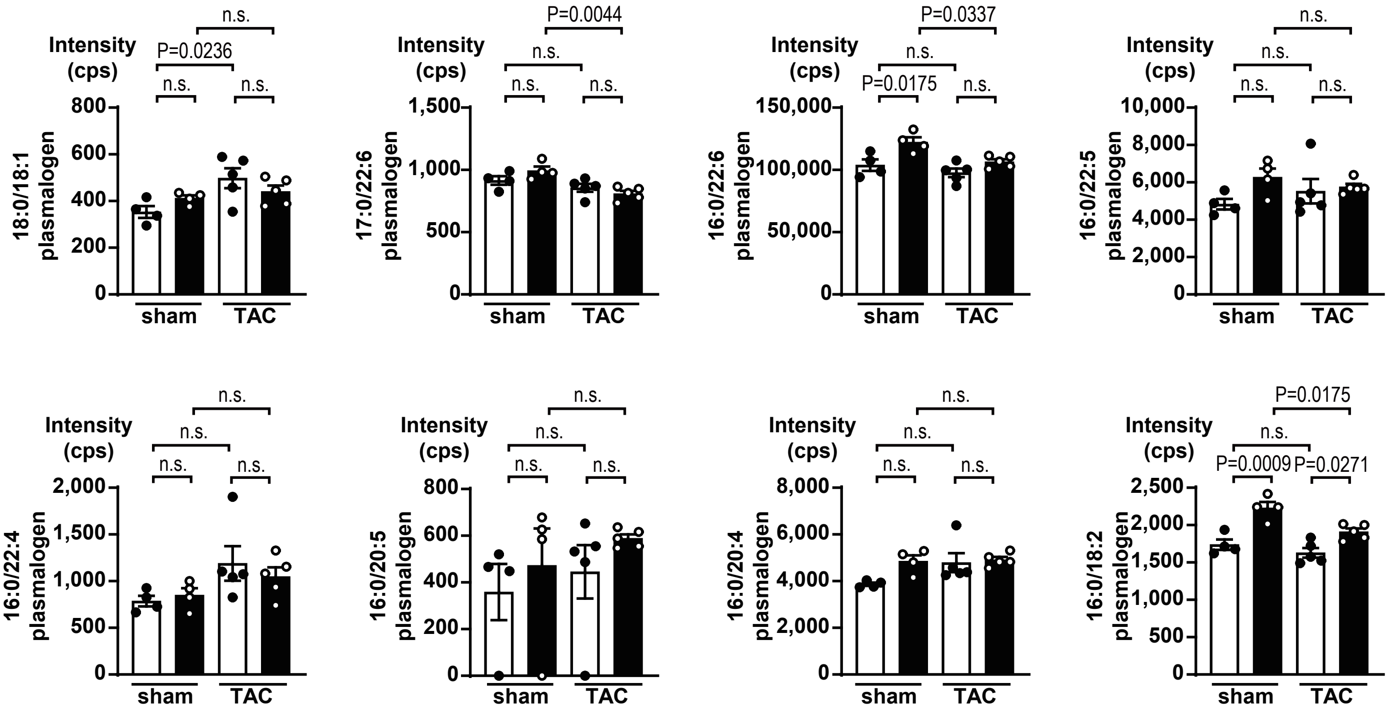

b

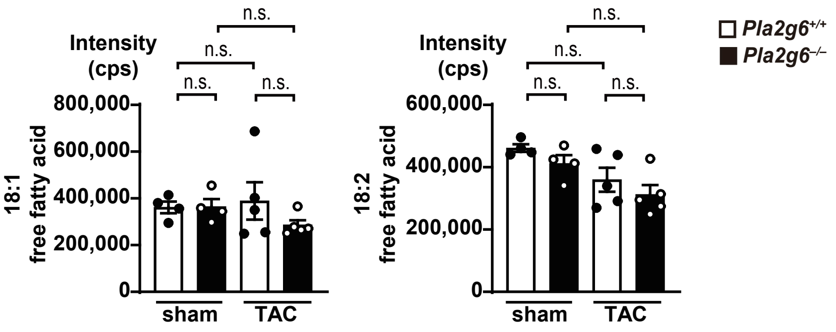

Supplementary Fig. 5 Continued

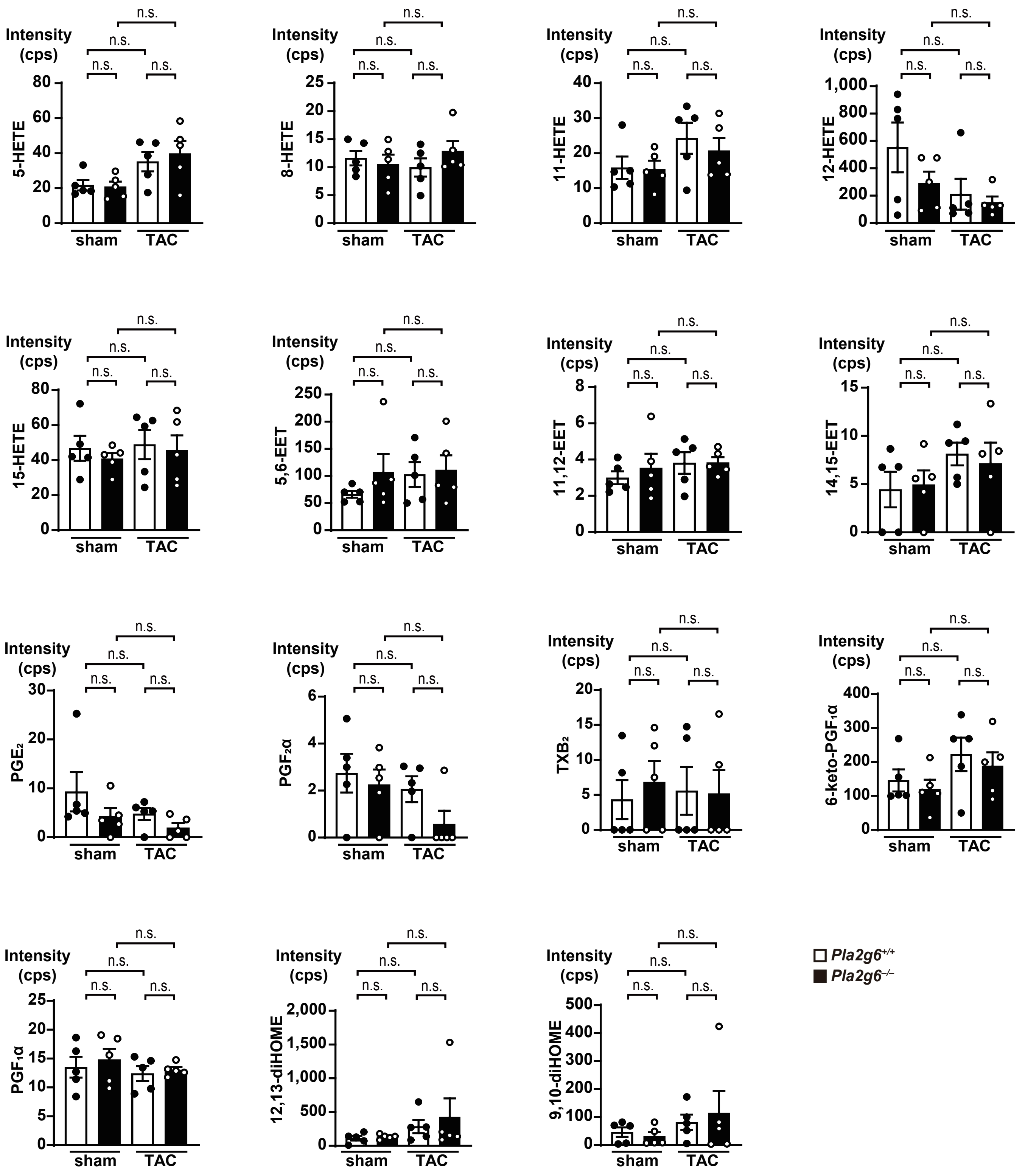

Supplementary Fig. 6: Quantity of lipid mediators associated with iPLA$_2$γ in mouse hearts after TAC operation.

Quantity of lipid mediators associated with iPLA$_2\gamma$ in mouse hearts after TAC operation. HETE, hydroxyeicosatetraenoic acid; EET, epoxyeicosatrienoic acid; PGE, prostaglandin E; PGF, prostaglandin F; TXB, thromboxane B; diHOME, dihydroxyoctadecenoic acid; HODE, hydroxyoctadecadienoic acid; oxo-ODE, oxo-octadecadienoic acid; EpOME, epoxyoctadecenoic acid; DiHDPA, dihydroxydocosapentaenoic acid; HDoHE, hydroxydocosahexaenoic acid; n = 5 (sham-operated *Pla2g6*$^{+/+}$), 5 (sham-operated *Pla2g6*$^{-/-}$), 5 (TAC-operated *Pla2g6*$^{+/+}$), and 5 (TAC-operated *Pla2g6*$^{-/-}$), biologically independent samples. Open and closed bars indicate *Pla2g6*$^{+/+}$ and *Pla2g6*$^{-/-}$ hearts, respectively. Data are expressed as the mean ± SEM. The data were evaluated by one-way ANOVA, followed by Tukey-Kramer's *post-hoc* test. Source data are provided as a Source Data file.

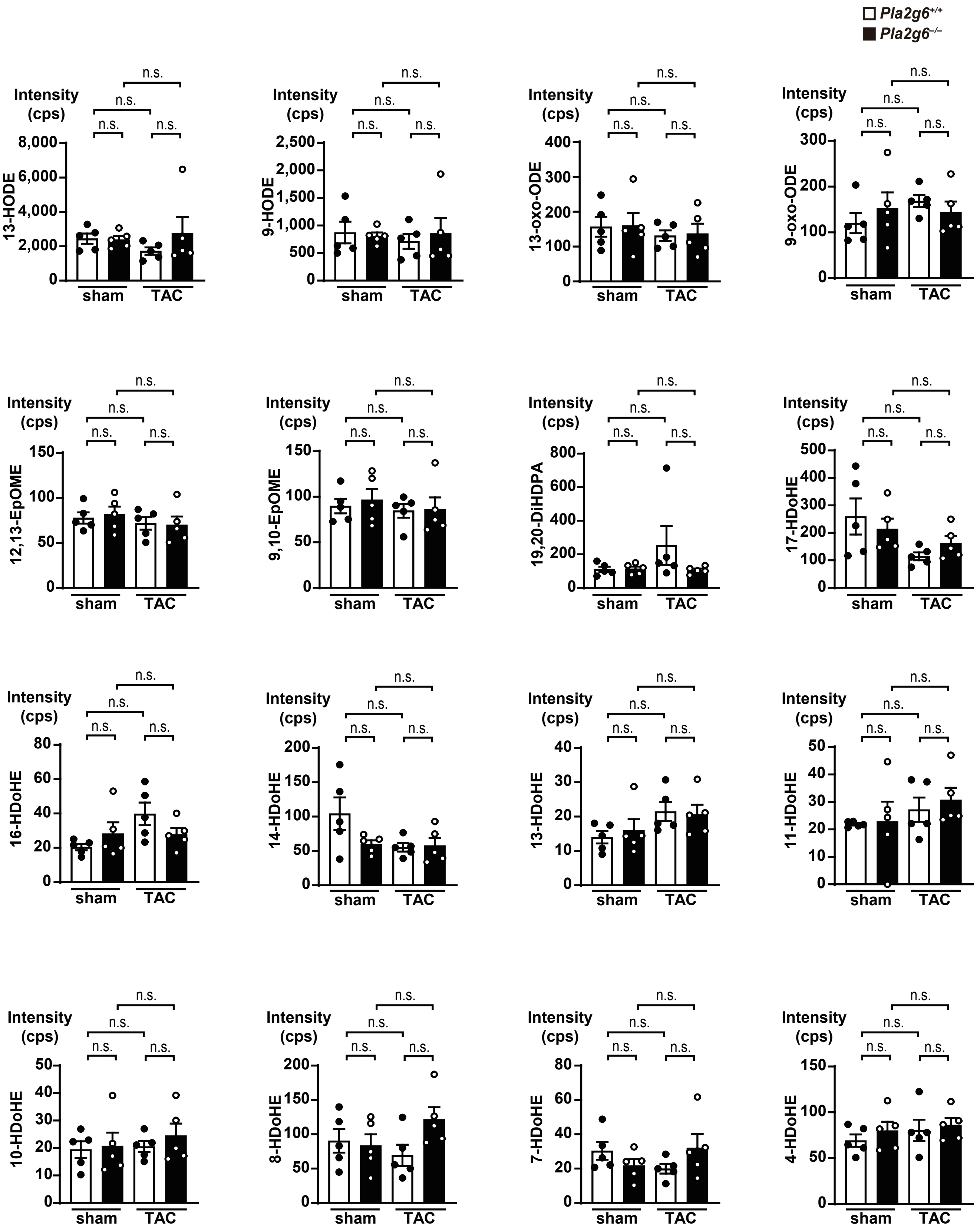

Supplementary Fig. 6 Continued.

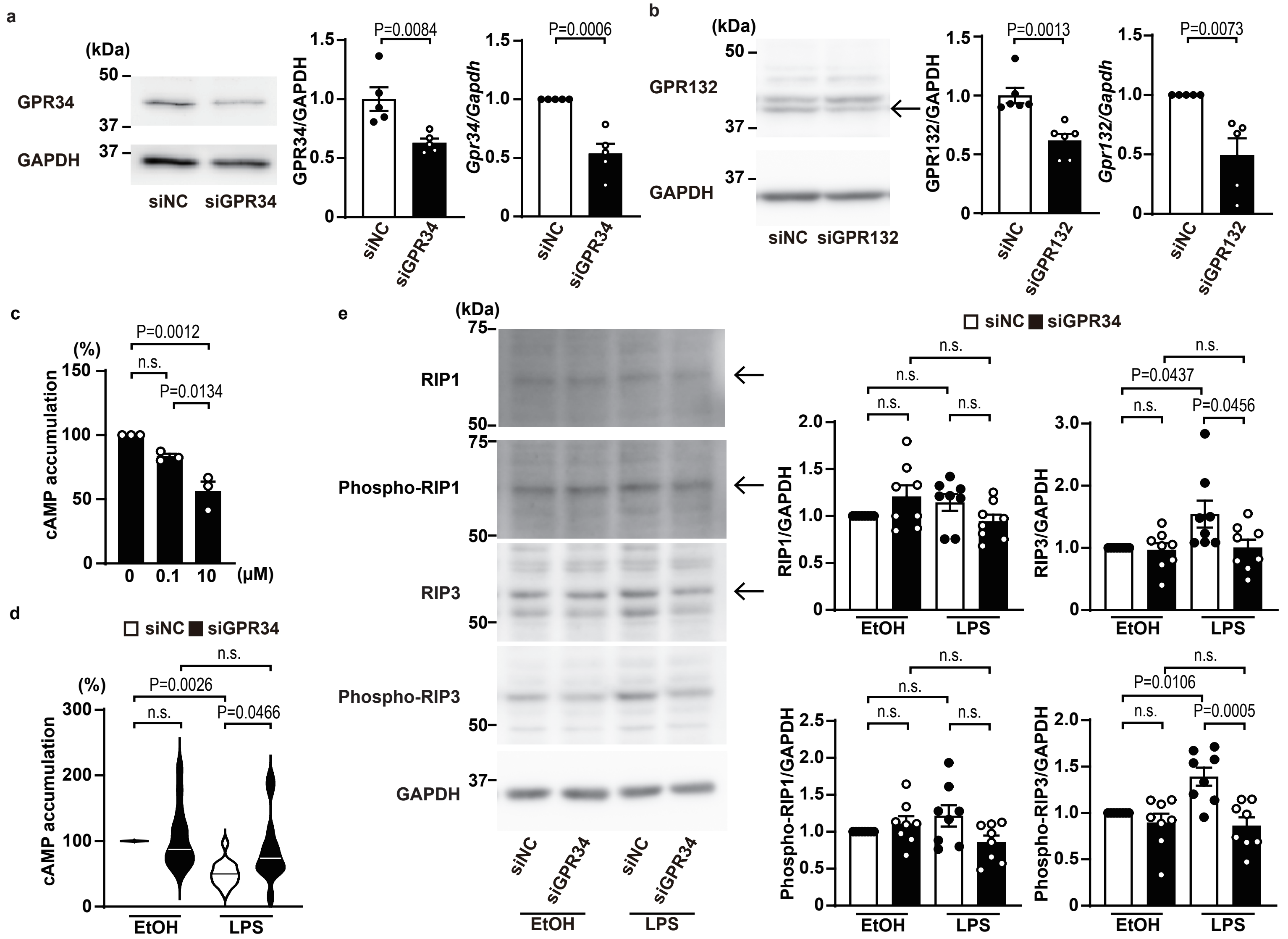

Supplementary Fig. 7: The efficiency of knockdown of lysophosphatidylserine receptors and signaling responses to lysophosphatidylserine through GPR34 in isolated rat neonatal cardiomyocytes.

a, **b** Isolated rat neonatal cardiomyocytes were transfected with siRNA targeted to GPR34 or GPR132 (siGPR34 or siGPR132) or with negative control siRNA (siNC). The left panels show the protein expression levels of GPR34 (**a**) and GPR132 (**b**), respectively. Densitometric analysis is shown in the middle panels. The right panels show the mRNA expression levels of *Gpr34* (**a**) and *Gpr132* (**b**), respectively. The average value of lysophosphatidylserine receptors normalized to GAPDH in siNC-transfected neonatal cardiomyocytes was set equal to 1. The *Gpr34*- or *Gpr132*-to-*Gapdh* ratio for siNC-transfected cells in each experiment was set equal to 1. The values are presented as the mean ± SEM. *n* = 5 independent experiments for both protein and mRNA expression in GPR34 group. *n* = 6 independent experiments for protein and *n* = 5 for mRNA expression in GPR132 group. The data were analyzed using two-sided unpaired Student's *t*-test. The samples derived from the same experiment and blots were processed in parallel. **c** Effect of 18:0 lysophosphatidylserine (LPS) on the accumulation of intracellular cyclic adenosine monophosphate (cAMP) in forskolin-stimulated cardiomyocytes. The cells were incubated with indicated concentrations of LPS. The cAMP level in the absence of LPS is defined as 100%. The percentage of intracellular cAMP is shown in the graph. *n* = 3, independent experiments. The data were evaluated by one-way ANOVA, followed by Tukey-Kramer's *post-hoc* test. **d** The effect of knockdown of GPR34 on intracellular cAMP in forskolin-stimulated cardiomyocytes with 10 μM LPS for 10 minutes. The cells were transfected with siGPR34 or siNC. Control sample was treated with 0.2% (v/v) ethanol (EtOH). The level in control samples transfected with siNC is defined as 100%. *n* = 14, independent experiments. The data were evaluated by one-way ANOVA, followed by Tukey-Kramer's *post-hoc* test. **e** Protein expression levels of RIP1 and RIP3 in cardiomyocytes after stimulation with 50 μM LPS. Densitometric analysis is shown in the right panels (*n* = 8, independent experiments). Control sample was treated with 1% (v/v) ethanol (EtOH). The value for siNC-transfected cells with EtOH treatment in each experiment was set equal to 1. The data were evaluated by one-way ANOVA, followed by Tukey-Kramer's *post-hoc* test. GAPDH was used as sample processing controls. The samples derived from the same experiment and blots were processed in parallel. Open and closed bars indicate siNC and siGPR34, respectively. In violin plots, solid lines show median. In bar graphs, data are expressed as the mean ± SEM. Source data are provided as a Source Data file.

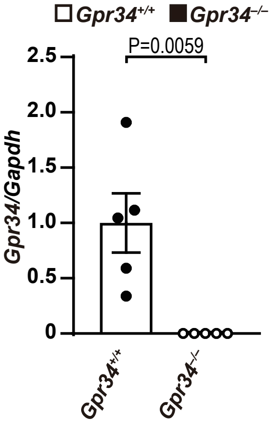

Supplementary Fig. 8: Expression level of *Gpr34* mRNA in GPR34-deficient mice.

Expression level of *Gpr34* mRNAs in *Gpr34*$^{+/+}$ and *Gpr34*$^{-/-}$ cardiomyocytes. *n* = 5, biologically independent samples. Data were normalized to the *Gapdh* content. The average value of *Gpr34*-to-*Gapdh* ratio in *Gpr34*$^{+/+}$ group was set equal to 1. Open and closed bars indicate *Gpr34*$^{+/+}$ and *Gpr34*$^{-/-}$, respectively. Data are expressed as the mean ± SEM. The data were analyzed using two-sided unpaired Student's *t*-test. Source data are provided as a Source Data file.

Supplementary Table 1. Echocardiographic parameters of *Pla2g6*$^{-/-}$ mice.

	Pla2g6$^{+/+}$ (n = 18)	*Pla2g6*$^{-/-}$ (n = 18)	P value
IVSd (mm)	0.84 ± 0.02	0.82 ± 0.02	0.398
LVIDd (mm)	2.67 ± 0.06	2.74 ± 0.07	0.502
LVIDs (mm)	1.22 ± 0.06	1.27 ± 0.06	0.535
LVPWd (mm)	0.84 ± 0.03	0.80 ± 0.03	0.283
FS (%)	54.6 ± 1.3	53.8 ± 1.3	0.667
HR (bpm)	683 ± 9	697 ± 8	0.264

IVSd, diastolic interventricle septal wall thickness; LVIDd, diastolic left ventricle internal dimension; LVIDs, systolic left ventricle internal dimension; LVPWd, diastolic left ventricle posterior wall thickness; FS, fractional shortening of left ventricle dimension; HR, heart rate. Two-sided unpaired Student's *t*-test was used. Values represent the mean ± SEM of data. Source data are provided as a Source Data file.

Supplementary Table 2. Physiological parameters of *Pla2g6*$^{-/-}$ mice.

	Pla2g6$^{+/+}$ (n = 13)	*Pla2g6*$^{-/-}$ (n = 13)	P value
Blood pressure (mmHg)	85 ± 2	85 ± 2	0.758
Body weight (g)	24.6 ± 0.7	24.6 ± 0.7	0.985
Tibia length (mm)	17.5 ± 0.1	17.6 ± 0.1	0.504
WH/Body weight (mg/g)	4.99 ± 0.10	5.14 ± 0.10	0.297
LV/Body weight (mg/g)	3.45 ± 0.07	3.49 ± 0.11	0.759
Lung/Body weight (mg/g)	5.24 ± 0.14	5.34 ± 0.18	0.653

WH/Body weight, whole heart weight-to-body weight ratio; LV/Body weight, left ventricle weight-to-body weight ratio; Lung/Body weight, lung weight-to-body weight ratio. Two-sided unpaired Student's *t*-test was used. Values represent the mean ± SEM of data. Source data are provided as a Source Data file.

Supplementary Table 3. Echocardiographic parameters of *Gpr34*$^{-/-}$ mice.

	Gpr34$^{+/+}$ (n = 18)	*Gpr34*$^{-/-}$ (n = 18)	P value
IVSd (mm)	0.74 ± 0.02	0.74 ± 0.02	0.984
LVIDd (mm)	3.28 ± 0.05	3.15 ± 0.05	0.074
LVIDs (mm)	1.49 ± 0.03	1.43 ± 0.02	0.122
LVPWd (mm)	0.78 ± 0.02	0.79 ± 0.04	0.842
FS (%)	54.5 ± 0.77	54.5 ± 0.56	0.963
HR (bpm)	719 ± 9	704 ± 10	0.273

IVSd, diastolic interventricle septal wall thickness; LVIDd, diastolic left ventricle internal dimension; LVIDs, systolic left ventricle internal dimension; LVPWd, diastolic left ventricle posterior wall thickness; FS, fractional shortening of left ventricle dimension; HR, heart rate. Two-sided unpaired Student's *t*-test was used. Values represent the mean ± SEM of data. Source data are provided as a Source Data file.

Supplementary Table 4. Primers for qPCR.

Species	Symbol	Forward primer sequence	Reverse primer sequence
Mouse	*Nppa*	TCGTCTTGGCCTTTTGGCT	TCCAGGTGGTCTAGCAGGTTCT
	Nppb	AAGTCCTAGCCAGTCTCCAGA	GAGCTGTCTCTGGGCCATTTC
	Col1a2	AGGCTGACACGAACTGAGGT	ATGCACATCAATGTGGAGGA
	Col3a1	ACAGCAAATTCACTTACACAGT	CTCATTGCCTTGCGTGTTT
	Il1b	CAACCAACAAGTGATATTCTCCATG	GATCCACACTCTCCAGCTGCA
	Il6	ACAACCACGGCCTTCCCTACTT	CACGATTTCCCAGAGAACATGTG
	Tnf	CTACCTTGTTGCCTCCTCTTT	GAGCAGAGGTTCAGTGATGTAG
	Il10	TATTCTAAGGCTGGCCACACT	GCTGCTGCAGGAATGATCAT
	Gpr34	ATATGCTACAACAGCCCGGA	GAACCGAAAGGCATGGTAAG
Rat	*Gpr34*	AATATGCCACGACAGCCCGG	TCGAAAGGCATGATAAGGCACGA
	Gpr132	GCAGGGCACTTGACAGAGCA	TGGCTGTAACGCTGTGGTGG
Mouse/Rat	*Gapdh*	ATGACAACTTTGTCAAGCTCATTT	GGTCCACCACCCTGTTGCT

Nppa, Natriuretic peptide type A; *Nppb*, Natriuretic peptide type B; *Col1a2*, Collagen, type I, alpha 2; *Col3a1*, Collagen, type III, alpha 1; *Il1b*, Interleukin 1 beta; *Il6*, Interleukin 6; *Tnf*, Tumor necrosis factor; *Il10*, Interleukin 10; *Gpr34*, G protein-coupled receptor 34; *Gpr132*, G protein-coupled receptor 132; *Gapdh*, Glyceraldehyde-3-phosphate dehydrogenase.

Separation Workflow

Created by https://lipidomicstandards.org, version v2.2.5

Overall study design

Title of the study	iPLA2beta		
Document creation date	05/31/2023	Corresponding Email	kinya.otsu@kcl.ac.uk
Principle investigator	Kinya Otsu	Is the workflow targeted or untargeted?	Untargeted
Institution	National Cerebral and Cardiovascular Center	Clinical	No

Lipid extraction

Extraction method	1-phase system	1-phase system	Methanol
pH adjustment	None	Were internal standards added prior extraction?	Yes

Analytical platform

Number of separation dimensions	One dimension	MS type	Q
Separation Type 1	LC	MS vendor	Agilent
Separation Mode 1	NP	Ion source	ESI
Separation window (1) for lipid analyte selection (±) in minutes	0	MS Level	MS1
RT verified by standard	Yes	Mass resolution for detected ion at MS1	Low resolution
CCS verified by standard	Yes	Resolution at MS1	Low
Separation of isobaric/isomeric interferece confirmed	Yes	Was/Were additional dimension/techniques used	No
Model for separation prediction	Yes		

Quality control

Blanks	Yes	Quality control	No
Type of Blanks	Extraction blank		

Method qualification and validation

Method validation	Yes	Precison	Yes
Lipid recovery	Yes	Accuracy	Yes
Dynamic quantification range	Yes	Guidelines followed	None
Limit of quantitation (LOQ)/Limit of detection (LOD)	Yes		

Reporting

Are reported raw data uploaded into repository?	Yes	Raw data upload	Yes
Are metadata available?	Yes	Additional comments	-
Summary data	Quantification and identification data		

Sample Descriptions

iPLA2beta knockout hearts / Mouse / Tissues (e.g., liver, heart, brain)

Perfusion	Yes	Storage time (month)	6
Provided information	Time to freeze (min), Storage time (month)	Additives	None
Temperature handling original sample	4-8 °C	Were samples stored under inert gas?	No
Instant sample preparation	No	Additional preservation methods	No
Time to freeze (min)	1	Biobank samples	No
Snap freezing in liquid N2	Yes	Sample homogenization	Yes
Storage temperature	-80 °C	Sample homogenization solvent	Methanol

Lipid Class Descriptions

1) LPS[M-H]- / Lipid identification

Lipid class	LPS	MS2 verified by standard	Yes
MS Level for identification	MS1, MS2	Background check at MS1	No
Identification level	Species level	Background check at MS2	No
Polarity mode	Negative	Check isomer overlap	No
Type of negative (precursor)ion	[M-H]-	Additional dimension/techniques	-
Fragments for identification		Lipid Identification Software	Homemade

Fragment name
-(C3H5NO2,87)
FA1(+O)

Isotope correction at MS1	No	Data manipulation	Centroiding
Isotope correction at MS2	No	Nomenclature for intact lipid molecule	No
MS1 verified by standard	No	Nomenclature for fragment ions	N/A

1) LPS[M-H]- / For additional separation methods/analytical dimension

Quantitative	No	Batch correction	No
Normalization to reference	No		

2) 5-HETE 8-HETE 11-HETE 12-HETE 15-HETE 5,6-EET 11,12-EET 14,15-EET PGE2 PGF2a TXB2 6-keto-PGF1a PGF1a 12,13-diHOME 9,10-diHOME 13-HODE 9-HODE 13-oxo-ODE 9-oxo-ODE 12,13-EpOME 9,10-EpOME 19,20-diHDoPE 17-HDoHE 16-HDoHE 14-HDoHE 13-HDoHE 11-HDoHE 10-HDoHE 8-HDoHE 7-HDoHE 4-HDoHE(MRM)[M-H]- / Lipid identification

Lipid class	5-HETE 8-HETE 11-HETE 12-HETE 15-HETE 5,6-EET 11,12-EET 14,15-EET PGE2 PGF2a TXB2 6-keto-PGF1a PGF1a 12,13-diHOME 9,10-diHOME 13-HODE 9-HODE 13-oxo-ODE 9-oxo-ODE 12,13-EpOME 9,10-EpOME 19,20-diHDoPE 17-HDoHE 16-HDoHE 14-HDoHE 13-HDoHE 11-HDoHE 10-HDoHE 8-HDoHE 7-HDoHE 4-HDoHE(MRM)	Did you presume assumptions for identification?	No
MS Level for identification	MS1, MS2	Check isomer overlap	No
Identification level	Molecular species level	RT verified by standard	Yes
Polarity mode	Negative	Separation of isobaric/isomeric interferece confirmed	Yes
Type of negative (precursor)ion	[M-H]-	Model for separation prediction	Yes
Fragments for identification	**Fragment name**<br>MRM(https://ars.els-cdn.com/content/image/1-s2.0-S2666166721001994-mmc2.xlsx)	Additional dimension/techniques	-
Isotope correction at MS1	No	Lipid Identification Software	MRM
Isotope correction at MS2	No	Data manipulation	Smoothing
MS1 verified by standard	Yes	Nomenclature for intact lipid molecule	No
MS2 verified by standard	Yes	Nomenclature for fragment ions	N/A
Background check at MS1	No	Further identification remarks	https://ars.els-cdn.com/content/image/1-s2.0-S2666166721001994-mmc2.xlsx
Background check at MS2	No		

2) 5-HETE 8-HETE 11-HETE 12-HETE 15-HETE 5,6-EET 11,12-EET 14,15-EET PGE2 PGF2a TXB2 6-keto-PGF1a PGF1a 12,13-diHOME 9,10-diHOME 13-HODE 9-HODE 13-oxo-ODE 9-oxo-ODE 12,13-EpOME 9,10-EpOME 19,20-diHDoPE 17-HDoHE 16-HDoHE 14-HDoHE 13-HDoHE 11-HDoHE 10-HDoHE 8-HDoHE 7-HDoHE 4-HDoHE(MRM)[M-H]- / Lipid quantification

Quantitative	No	Batch correction	No
Normalization to reference	Yes	Further quantification remarks	https://www.sciencedirect.com/science/article/pii/S2666166721001994?via%3Dihub

3) PS[M-H]- / Lipid identification

Lipid class	PS	MS2 verified by standard	Yes
MS Level for identification	MS1, MS2	Background check at MS1	No
Identification level	Species level	Background check at MS2	No
Polarity mode	Negative	Check isomer overlap	No
Type of negative (precursor)ion	[M-H]-	Additional dimension/techniques	-
Fragments for identification		Lipid Identification Software	Homemade

Fragment name
-FA1(-H)-(C3H5NO2)
FA1(+O)
FA2(+O)
-FA2(-H)-(C3H5NO2)
-(C3H5NO2,87)

Isotope correction at MS1	No	Data manipulation	Centroiding
Isotope correction at MS2	No	Nomenclature for intact lipid molecule	No
MS1 verified by standard	No	Nomenclature for fragment ions	N/A

3) PS[M-H]- / For additional separation methods/analytical dimension

Quantitative	No	Batch correction	No
Normalization to reference	No		

4) PC[M+CH3COO]- / Lipid identification

Lipid class	PC	MS2 verified by standard	Yes
MS Level for identification	MS1, MS2	Background check at MS1	No
Identification level	Species level	Background check at MS2	No
Polarity mode	Negative	Check isomer overlap	No
Type of negative (precursor)ion	[M+CH3COO]-	Additional dimension/techniques	-
Fragments for identification		Lipid Identification Software	Homemade

Fragment name
-FA1(+HO)-(CH3-+CH3COO)
FA1(+O)
FA2(+O)
-FA2(+HO)-(CH3-+CH3COO)
-(CH3+CH3COO)

Isotope correction at MS1	No	Data manipulation	Centroiding
Isotope correction at MS2	No	Nomenclature for intact lipid molecule	No
MS1 verified by standard	No	Nomenclature for fragment ions	N/A

4) PC[M+CH3COO]- / For additional separation methods/analytical dimension

Quantitative	No	Batch correction	No
Normalization to reference	No		

5) PI[M-H]- / Lipid identification

Lipid class	PI	MS2 verified by standard	Yes
MS Level for identification	MS1, MS2	Background check at MS1	No
Identification level	Species level	Background check at MS2	No
Polarity mode	Negative	Check isomer overlap	No
Type of negative (precursor)ion	[M-H]-	Additional dimension/techniques	-
Fragments for identification		Lipid Identification Software	Homemade

Fragment name
FA1(+O)
FA2(+O)
-FA1(+HO)
-FA1(-H)
-FA2(+HO)
-FA2(-H)
HG(PI,241)

Isotope correction at MS1	No	Data manipulation	Centroiding
Isotope correction at MS2	No	Nomenclature for intact lipid molecule	No
MS1 verified by standard	No	Nomenclature for fragment ions	N/A

5) PI[M-H]- / For additional separation methods/analytical dimension

Quantitative	No	Batch correction	No
Normalization to reference	No		

6) PG[M-H]- / Lipid identification

Lipid class	PG	MS2 verified by standard	Yes
MS Level for identification	MS1, MS2	Background check at MS1	No
Identification level	Species level	Background check at MS2	No
Polarity mode	Negative	Check isomer overlap	No
Type of negative (precursor)ion	[M-H]-	Additional dimension/techniques	-
Fragments for identification		Lipid Identification Software	Homemade

Fragment name
FA1(+O)
FA2(+O)
-FA1(+HO)
-FA2(+HO)
GP(153)
HG(PG,227)

Isotope correction at MS1	No	Data manipulation	Centroiding
Isotope correction at MS2	No	Nomenclature for intact lipid molecule	No
MS1 verified by standard	No	Nomenclature for fragment ions	N/A

6) PG[M-H]- / For additional separation methods/analytical dimension

Quantitative	No	Batch correction	No
Normalization to reference	No		

7) BMP[M-H]- / Lipid identification

Lipid class	BMP	MS2 verified by standard	Yes
MS Level for identification	MS1, MS2	Background check at MS1	No
Identification level	Species level	Background check at MS2	No
Polarity mode	Negative	Check isomer overlap	No
Type of negative (precursor)ion	[M-H]-	Additional dimension/techniques	-
Fragments for identification		Lipid Identification Software	Homemade

Fragment name
FA1(+O)
FA2(+O)
-FA1(+HO)
-FA2(+HO)
GP(153)
HG(GPG,227)

Isotope correction at MS1	No	Data manipulation	Centroiding
Isotope correction at MS2	No	Nomenclature for intact lipid molecule	No
MS1 verified by standard	No	Nomenclature for fragment ions	N/A

7) BMP[M-H]- / For additional separation methods/analytical dimension

Quantitative	No	Batch correction	No
Normalization to reference	No		

8) PE P[M-H]- / Lipid identification

Lipid class	PE P	MS2 verified by standard	Yes
MS Level for identification	MS1, MS2	Background check at MS1	No
Identification level	Species level	Background check at MS2	No
Polarity mode	Negative	Check isomer overlap	No
Type of negative (precursor)ion	[M-H]-	Additional dimension/techniques	-
Fragments for identification		Lipid Identification Software	Homemade

Fragment name
FA2(+O)
-FA2(+HO)
HG(PE,196)
FA O-[xx:x]

Isotope correction at MS1	No	Data manipulation	Centroiding
Isotope correction at MS2	No	Nomenclature for intact lipid molecule	No
MS1 verified by standard	No	Nomenclature for fragment ions	N/A

8) PE P[M-H]- / For additional separation methods/analytical dimension

Quantitative	No	Batch correction	No
Normalization to reference	No		

9) FA[M-H]- / Lipid identification

Lipid class	FA	Background check at MS1	No
MS Level for identification	MS1	Check isomer overlap	No
Identification level	Species level	Additional dimension/techniques	-
Polarity mode	Negative	Lipid Identification Software	Homemade
Type of negative (precursor)ion	[M-H]-	Data manipulation	Centroiding
Isotope correction at MS1	No	Nomenclature for intact lipid molecule	No
MS1 verified by standard	No		

9) FA[M-H]- / For additional separation methods/analytical dimension

Quantitative	No	Batch correction	No
Normalization to reference	No		

10) BMP(Pos reference)[M+NH4]+ / Lipid identification

Lipid class	BMP(Pos reference)	MS2 verified by standard	Yes
MS Level for identification	MS1, MS2	Background check at MS1	No
Identification level	Species level	Background check at MS2	No
Polarity mode	Positive	Check isomer overlap	No
Type of positive (precursor)ion	[M+NH4]+	Additional dimension/techniques	-
Fragments for identification		Lipid Identification Software	Homemade

Fragment name
FA1(+C3H4O)
FA2(+C3H4O)

Isotope correction at MS1	No	Data manipulation	Centroiding
Isotope correction at MS2	No	Nomenclature for intact lipid molecule	No
MS1 verified by standard	No	Nomenclature for fragment ions	N/A

10) BMP(Pos reference)[M+NH4]+ / For additional separation methods/analytical dimension

Quantitative	No	Batch correction	No
Normalization to reference	No		

11) PG(Pos reference)[M+NH4]+ / Lipid identification

Lipid class	PG(Pos reference)	MS2 verified by standard	Yes
MS Level for identification	MS1, MS2	Background check at MS1	No
Identification level	Species level	Background check at MS2	No
Polarity mode	Positive	Check isomer overlap	No
Type of positive (precursor)ion	[M+NH4]+	Additional dimension/techniques	-
Fragments for identification		Lipid Identification Software	Homemade

Fragment name
-HG(PG,172)
FA1(+C3H4O)
FA2(+C3H4O)

Isotope correction at MS1	No	Data manipulation	Centroiding
Isotope correction at MS2	No	Nomenclature for intact lipid molecule	No
MS1 verified by standard	No	Nomenclature for fragment ions	N/A

11) PG(Pos reference)[M+NH4]+ / For additional separation methods/analytical dimension

Quantitative	No	Batch correction	No
Normalization to reference	No		

Supplementary figure 1b

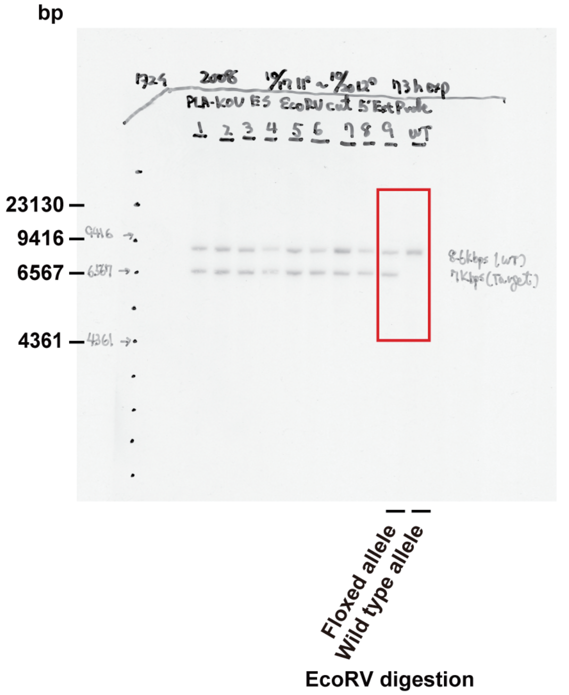

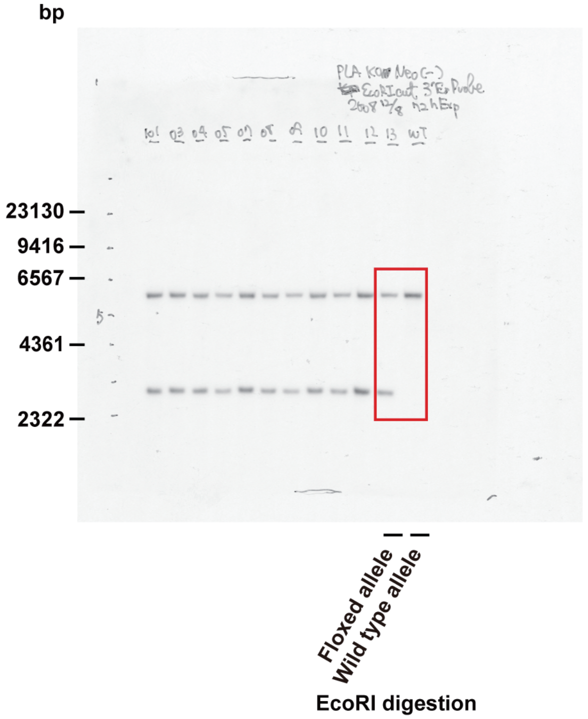

Supplementary figure 1c

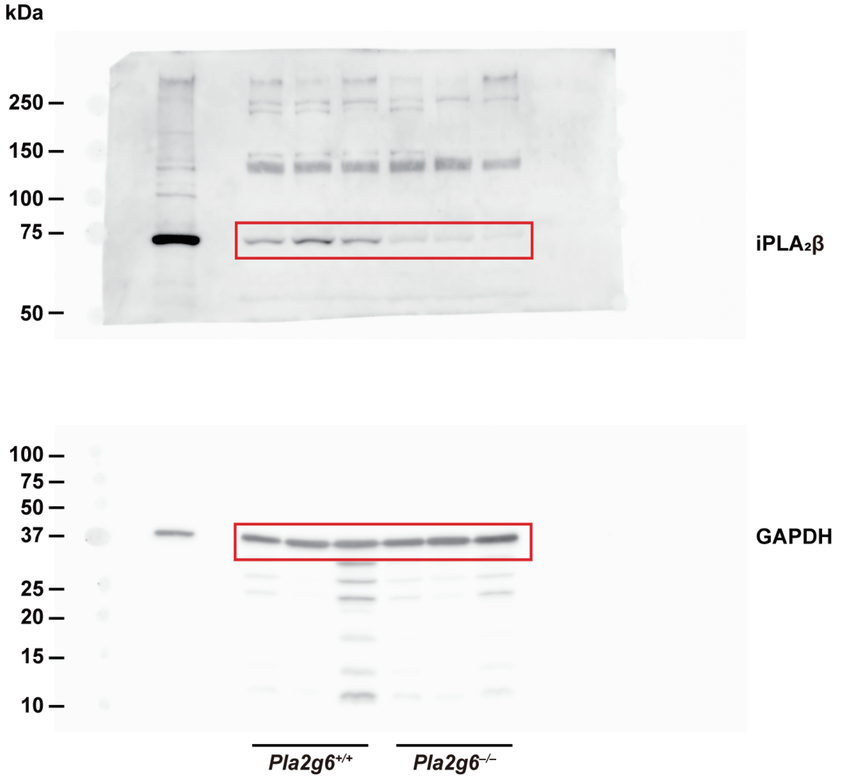

Uncropped scans of blots in supplementary figures.

Red squares show the part of cropped area.

Supplementary figure 1d

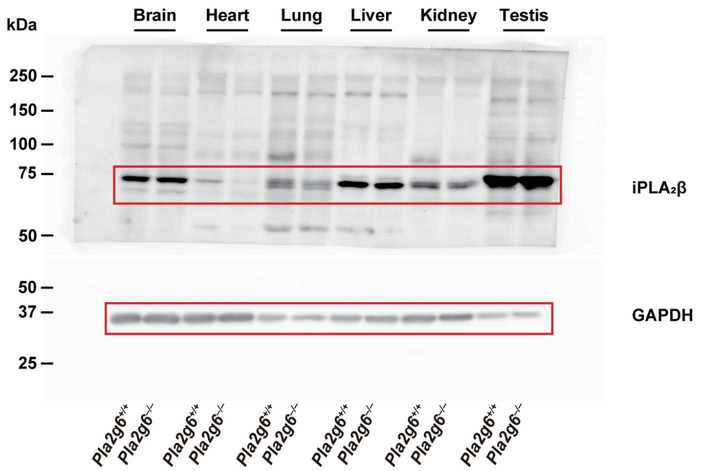

Supplementary figure 1e

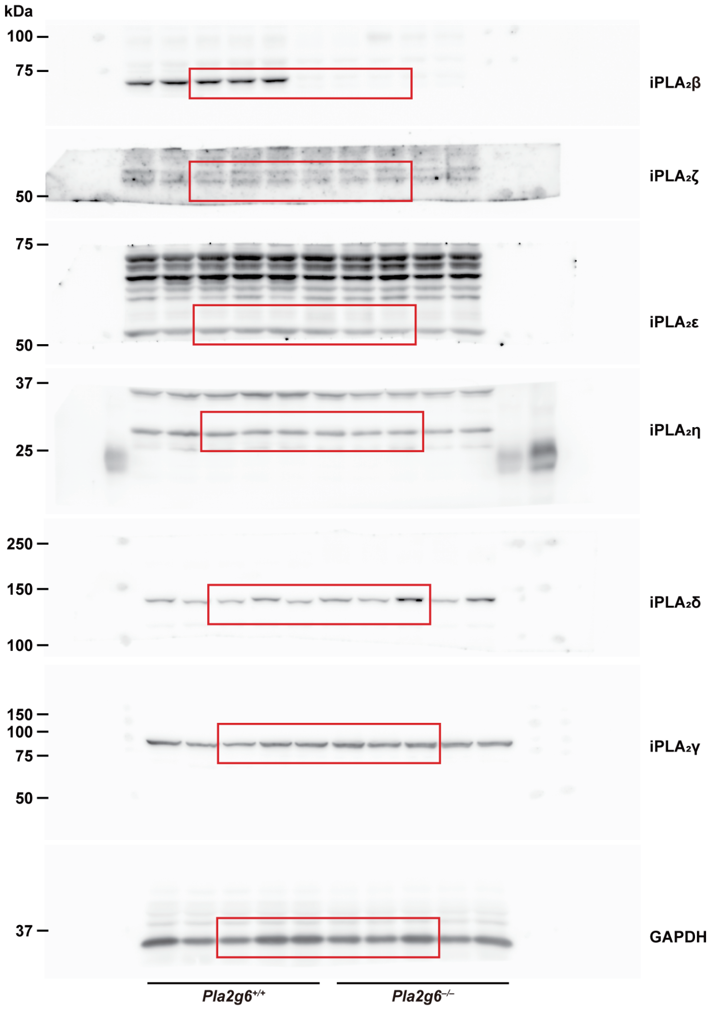

Supplementary figure 1f

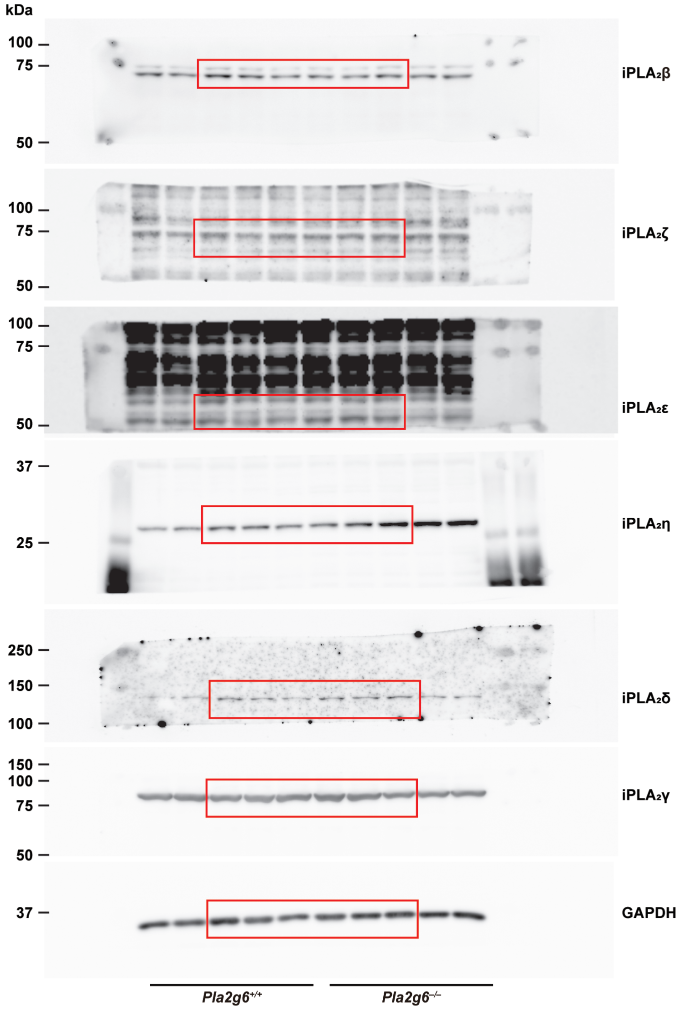

Uncropped scans of blots in supplementary figures. (Continued)

Supplementary figure 2a

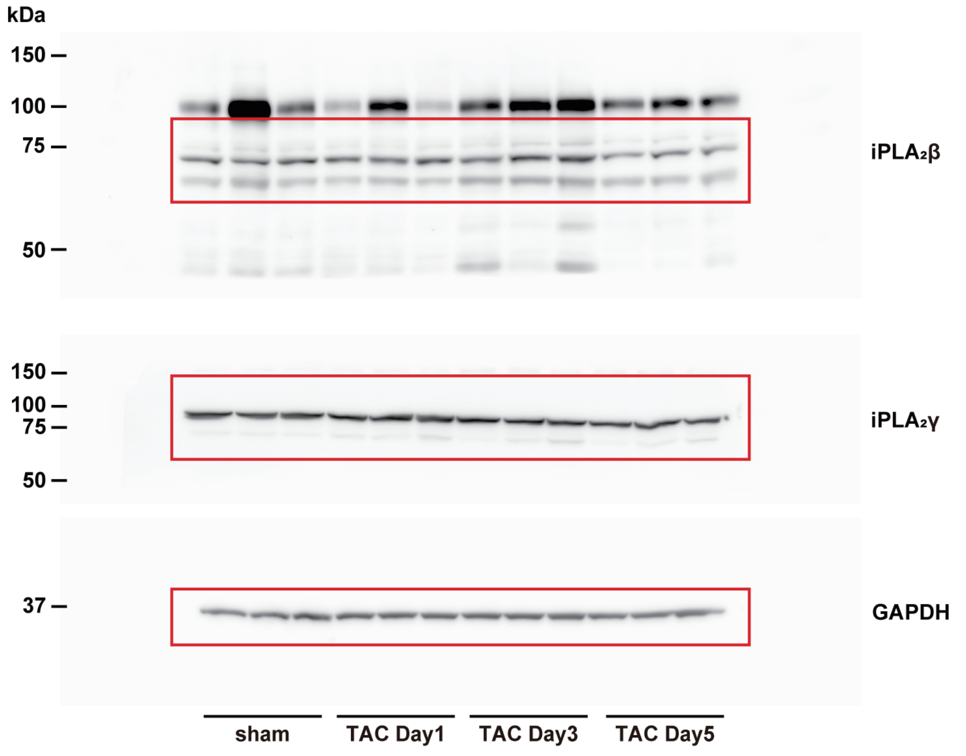

Supplementary figure 2b

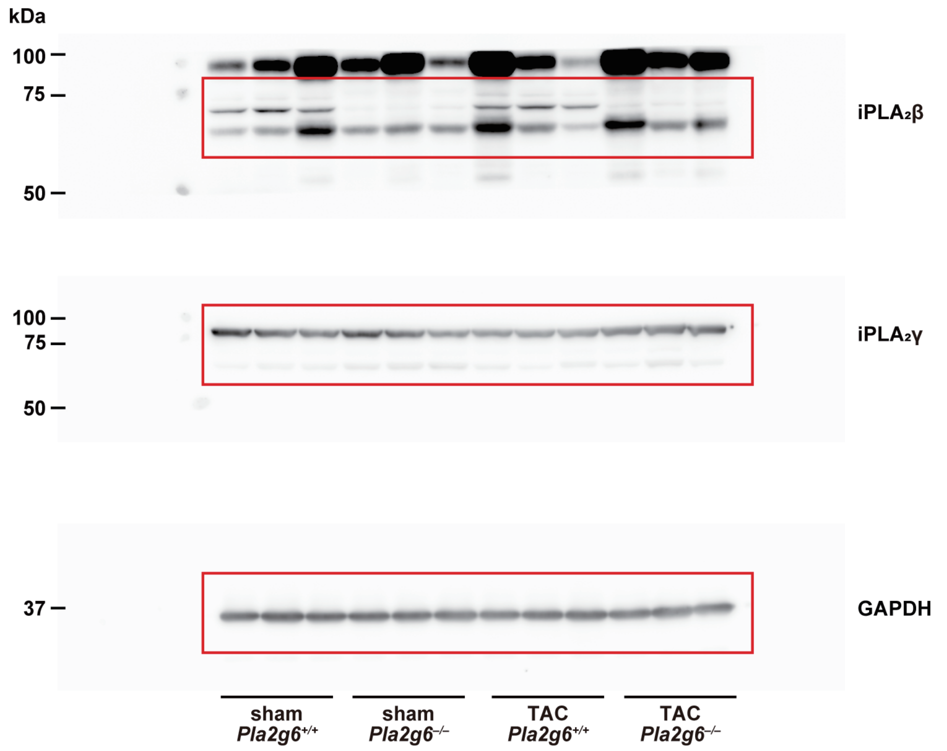

Uncropped scans of blots in supplementary figures. (Continued)

Supplementary figure 7a

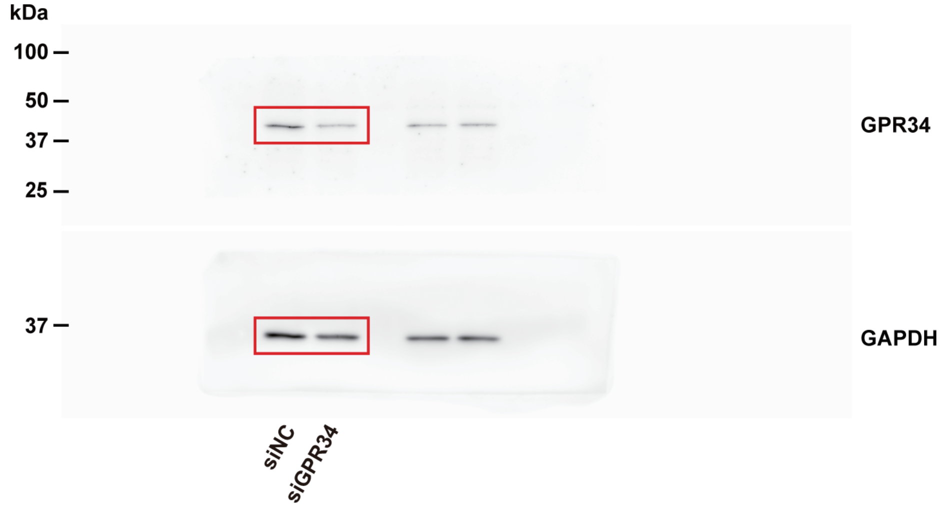

Supplementary figure 7b

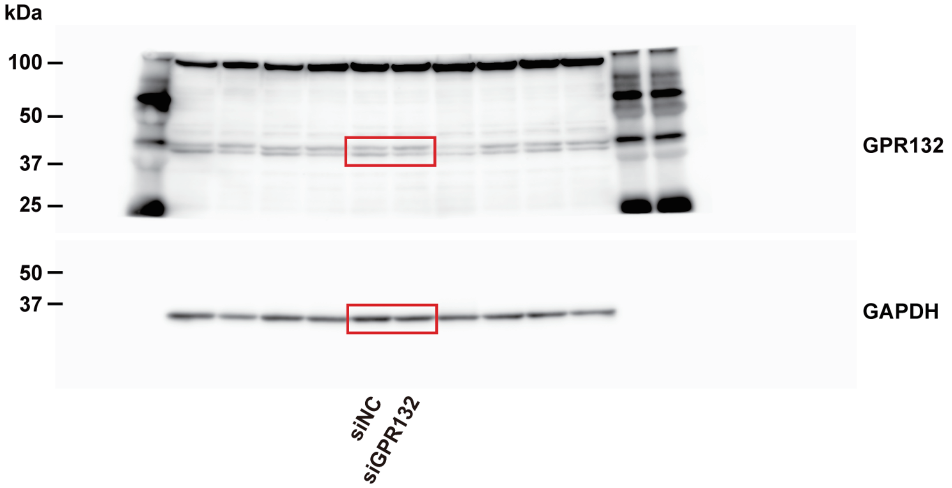

Uncropped scans of blots in supplementary figures. (Continued)

Supplementary figure 7e

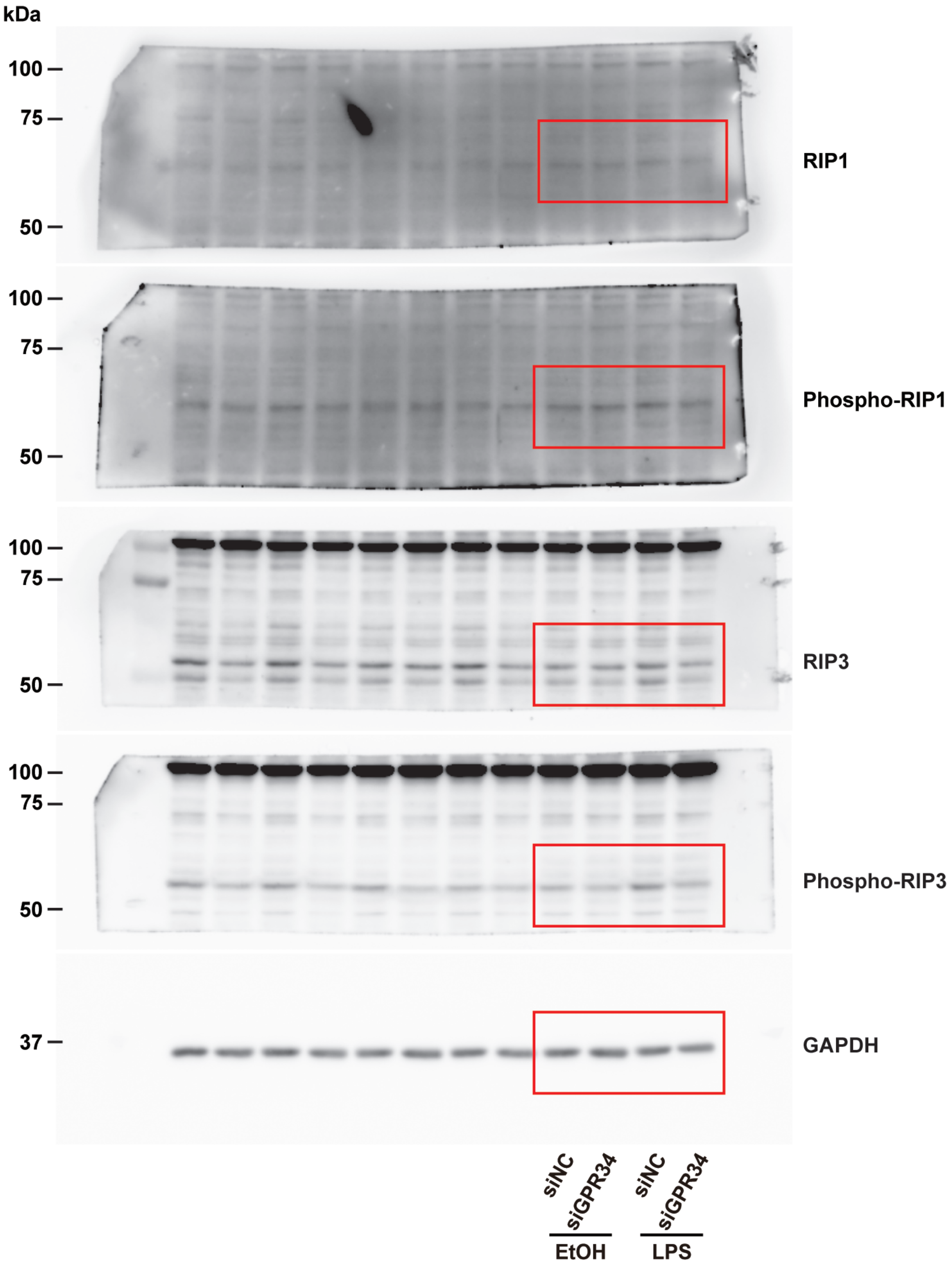

Uncropped scans of blots in supplementary figures. (Continued)
