# Supplementary material for: Lysophosphatidylserine induces necrosis in pressure overloaded male mouse hearts via G protein coupled receptor 34
Source: Nat Commun. 2023 Jul 31;14:4494. doi: 10.1038/s41467-023-40201-4 (PMC10390482; doi:10.1038/s41467-023-40201-4)
Supplement: Supplementary file 2 — Reporting Summary [file 41467_2023_40201_MOESM2_ESM.pdf]

nature portfolio

Corresponding author(s): Kinya Otsu

Last updated by author(s): Jun 1, 2023

Reporting Summary

Nature Portfolio wishes to improve the reproducibility of the work that we publish. This form provides structure for consistency and transparency in reporting. For further information on Nature Portfolio policies, see our Editorial Policies and the Editorial Policy Checklist.

Statistics

For all statistical analyses, confirm that the following items are present in the figure legend, table legend, main text, or Methods section.

n/a	Confirmed	
☐	☒	The exact sample size (*n*) for each experimental group/condition, given as a discrete number and unit of measurement
☐	☒	A statement on whether measurements were taken from distinct samples or whether the same sample was measured repeatedly
☐	☒	The statistical test(s) used AND whether they are one- or two-sided<br>*Only common tests should be described solely by name; describe more complex techniques in the Methods section.*
☐	☒	A description of all covariates tested
☐	☒	A description of any assumptions or corrections, such as tests of normality and adjustment for multiple comparisons
☐	☒	A full description of the statistical parameters including central tendency (e.g. means) or other basic estimates (e.g. regression coefficient) AND variation (e.g. standard deviation) or associated estimates of uncertainty (e.g. confidence intervals)
☐	☒	For null hypothesis testing, the test statistic (e.g. *F*, *t*, *r*) with confidence intervals, effect sizes, degrees of freedom and *P* value noted<br>*Give P values as exact values whenever suitable.*
☒	☐	For Bayesian analysis, information on the choice of priors and Markov chain Monte Carlo settings
☒	☐	For hierarchical and complex designs, identification of the appropriate level for tests and full reporting of outcomes
☒	☐	Estimates of effect sizes (e.g. Cohen's *d*, Pearson's *r*), indicating how they were calculated

Our web collection on statistics for biologists contains articles on many of the points above.

Software and code

Policy information about availability of computer code

Data collection	Echocardiogram : SONOS-4500 (Philips Medical Systems)<br>Blood pressure: Model MK-2000 (Muromachi Kikai)<br>qRT-PCR data collection: QuantStudio 7 Flex (Applied Biosystems)<br>IHC-image scan: BZX710 (Keyence), BZX810 (Keyence), or FV-1000D (Olympus)<br>Cell viability and cAMP assay: SH-9000lab (Hitachi High-Tech Science Corporation)<br>Western Blot analysis: ImageQuant LAS4000mini (Cytiva)<br>Lipidomics analysis: Analyst TF v1.8.1 and Analyst Software v1.6 (SCIEX)
Data analysis	Plots and statistics: Graphpadprism v8.4.3 (GraphPad Software)<br>Quantification of qRT-PCR data: QuantStudio Realtime PCR software v1.3 (Applied Biosystems)<br>IHC-image quantification analysis: ImageJ v1.51j8 (National Institutes of Health)<br>Western Blot analysis: ImageQuantTL v7.0 (Cytiva)<br>Lipidomics analysis: MultiQuant software v2.0 (SCIEX), SCIEX MS data converter v1.3 beta (SCIEX) and 2DICAL v0.91 (Mitsui Knowledge Industry)

For manuscripts utilizing custom algorithms or software that are central to the research but not yet described in published literature, software must be made available to editors and reviewers. We strongly encourage code deposition in a community repository (e.g. GitHub). See the Nature Portfolio guidelines for submitting code & software for further information.

Data

Policy information about availability of data

All manuscripts must include a data availability statement. This statement should provide the following information, where applicable:

- Accession codes, unique identifiers, or web links for publicly available datasets
- A description of any restrictions on data availability
- For clinical datasets or third party data, please ensure that the statement adheres to our policy

All data supporting the findings of this study are available within the article and its Supplementary Information files. Lipidomics raw data are also available from an online resource, MetaboBank (https://mb2.ddbj.nig.ac.jp/study/MTBKS202.html). Source data are provided with this manuscript.

Research involving human participants, their data, or biological material

Policy information about studies with human participants or human data. See also policy information about sex, gender (identity/presentation), and sexual orientation and race, ethnicity and racism.

Reporting on sex and gender	No human participants were used in this study.
Reporting on race, ethnicity, or other socially relevant groupings	No human participants were used in this study.
Population characteristics	No human participants were used in this study.
Recruitment	No human participants were used in this study.
Ethics oversight	No human participants were used in this study.

Note that full information on the approval of the study protocol must also be provided in the manuscript.

Field-specific reporting

Please select the one below that is the best fit for your research. If you are not sure, read the appropriate sections before making your selection.

☒ Life sciences ☐ Behavioural & social sciences ☐ Ecological, evolutionary & environmental sciences

For a reference copy of the document with all sections, see nature.com/documents/nr-reporting-summary-flat.pdf

Life sciences study design

All studies must disclose on these points even when the disclosure is negative.

Sample size	No calculations were made to predetermine sample size. Sample sizes and the number of independent experiments were chosen based on our previous experience and literature review to provide a sufficient sample size for statistical analysis and to obtain strong inferences of meaningful conclusions (Nakai A et al. Nat Med. 2007, PMID: 17450150; Oka T et al. Nature. 2012, PMID: 22535248).<br>For the animal study, a sample size of at least seven biologically independent samples was chosen. A survival study was performed with a sample size of at least 50 for TAC-operated groups and at least 13 for sham-operated groups.<br>For each experiment using in vivo samples (immunohistological analysis, qRT-PCR, serum troponin T test, southern and western blotting), results were confirmed with at least five samples per group. For lipidomics analysis, at least four per group were chosen in order to provide a sufficient sample size for statistical analysis.<br>For each biochemical analysis using in vitro samples (CTB assay, western blot, qRT-PCR, and cAMP assay), we performed a minimum of three independent experiments. For immunocytological analysis, we quantified at least 5 cells each from 10 individual fields of view (50 cells total).
Data exclusions	No data were excluded.
Replication	All in vitro experiments were replicated 3 or more times. For mouse tissue studies, experiments were replicated in 4 or more independent biological samples and in accordance with the 3R.
Randomization	Age/body weight/sex matched litter mates were assigned to groups by genotype.<br>In vitro samples were randomly allocated into experimental groups.
Blinding	Investigator was blinded for in vivo and in vitro experiments.

Reporting for specific materials, systems and methods

We require information from authors about some types of materials, experimental systems and methods used in many studies. Here, indicate whether each material, system or method listed is relevant to your study. If you are not sure if a list item applies to your research, read the appropriate section before selecting a response.

Materials & experimental systems

n/a	Involved in the study
☐	☒ Antibodies
☒	☐ Eukaryotic cell lines
☒	☐ Palaeontology and archaeology
☐	☒ Animals and other organisms
☒	☐ Clinical data
☒	☐ Dual use research of concern
☒	☐ Plants

Methods

n/a	Involved in the study
☒	☐ ChIP-seq
☒	☐ Flow cytometry
☒	☐ MRI-based neuroimaging

Antibodies

Antibodies used

Western blotting
Primary antibodies;
Anti-iPLA2β, Santa Cruz Biotechnology, sc376563, Product Clone Name; D-4 (1:100 dilution)
Anti-GAPDH, FUJIFILM Wako Pure Chemical Corporation, 016-25523, Product Clone Name; 5A12 (1:1000 dilution)
Anti-iPLA2ζ, Abnova, MAB3210, Product Clone Name; AT2G2 (1:1000 dilution)
Anti-iPLA2ε, Everest biotech, EB08402 (1:1000 dilution)
Anti-iPLA2η, Protein tech, 25469-1-AP (1:500 dilution)
Anti-iPLA2δ, Nobus bio, NBP1-74214 (1:500 dilution)
Anti-iPLA2γ, Thermo Fisher Scientific, PA5-49993 (1:1000 dilution)
Anti-GPR34, Thermo Fisher Scientific, PA5-45717 (1:1000 dilution)
Anti-GPR132, Protein tech, 17026-1-AP (1:1000 dilution)
Anti-RIP1, Cell Signaling Technology, #3493, Product Clone Name;D94C12 (1:1000 dilution)
Anti-Phospho-RIP1, Cell Signaling Technology, #31122 (1:1000 dilution)
Anti-RIP3, Nobus bio, NBP1-77299 (1:1000 dilution)
Anti-Phospho-RIP3, Abcam, ab195117, Product Clone Name; EPR9516(N)-25 (1:1000 dilution)
Secondary antibodies;
Anti-Mouse IgG HRP Linked Whole Ab, Cytiva, NA931 (1:10000 dilution)
Anti-Rabbit IgG HRP Linked Whole Ab, Cytiva, NA934 (1:10000 dilution)
Immunohistochemistry:
Anti-CD45, Biolegend, 103101, Product Clone Name; 30-F11 (1:100 dilution)
Anti-CD68, Bio-Rad, MCA1957T, Product Clone Name; FA-11 (1:100 dilution)
Anti-Ly-6G/Ly-6C, BD Pharmacy, 550291, Product Clone Name; RB6-8C5 (1:100 dilution)
Anti-CD3, Abcam, ab16669, Product Clone Name; SP7 (1:100 dilution)
Anti-HMGB1, Abcam, ab18256 (1:100 dilution)
Anti-α-sarcomeric actin, Thermo Fisher Scientific, A2172, Product Clone Name; 5C5 (1:500 dilution)
Secondary antibodies;
Anti-Rat IgG (H+L), Biotinylated Ab, Vector Laboratory, BA-4001 (1:200 dilution)
Anti-Rabbit IgG (H+L), Biotinylated Ab, Vector Laboratory, BA-1000 (1:200 dilution)
Anti-Rabbit IgG (H+L), Alexa Fluor™ 488 conjugated Ab, Invitrogen, A11034 (1:100 dilution)
Anti-Mouse IgM, Texas Red® conjugated Ab, Vector Laboratory, TI2020 (1:10 dilution)

Validation

All antibodies in this study were commercially purchased and have been validated by the vendors for species and application.
Validation data are available from the respective vendor's respective websites.

Anti-iPLA2β; https://www.scbt.com/p/group-vi-ipla2-antibody-d-4?productCanUrl=group-vi-ipla2-antibody-d-4&_requestid=2462021
Species specificity: Mouse, Rat, Human
Applications: Western blotting, Immunoprecipitation, Immunofluorescence, Enzyme-linked immuno-sorbent assay

Anti-GAPDH; https://labchem-wako.fujifilm.com/us/product/detail/W01W0101-2552.html
Species specificity: Human, African Green Monkey
Applications: Western blotting, Immunoprecipitation

Anti-iPLA2ζ; https://www.abnova.com/products/products_detail.asp?catalog_id=MAB3210
Species specificity: Human, Mouse
Applications: Enzyme-linked immuno-sorbent assay , Immunohistochemistry, Western blotting

Anti-iPLA2ε; https://everestbiotech.com/product/goat-anti-pnpla3-adiponutrin-antibody/
Species specificity: Human, Mouse, Rat
Applications: Enzyme-linked immuno-sorbent assay , Immunohistochemistry, Western blotting

Anti-iPLA2η; https://www.ptglab.co.jp/products/PNPLA4-Antibody-25469-1-AP.htm
Species specificity: Human, Mouse
Applications: Western blotting, Immunoprecipitation, Enzyme-linked immuno-sorbent assay

Anti-iPLA2δ; https://www.novusbio.com/products/pnpla6-antibody_nbp1-74214

Species specificity: Mouse
Applications: Western blotting

Anti-iPLA2γ; https://www.thermofisher.com/antibody/product/PNPLA8-Antibody-Polyclonal/PA5-49993
Species specificity: Human, Mouse
Applications: Western blotting

Anti-GPR34; https://www.thermofisher.com/antibody/product/GPR34-Antibody-Polyclonal/PA5-45717
Species specificity: Human, Mouse
Applications: Western blotting

Anti-GPR132; https://www.ptglab.co.jp/products/GPR132-Antibody-17026-1-AP.htm
Species specificity: Human, Mouse, Rat
Applications: Enzyme-linked immuno-sorbent assay , Immunohistochemistry, Western blotting

Anti-RIP1; https://www.cellsignal.jp/products/primary-antibodies/rip-d94c12-xp-rabbit-mab/3493
Species specificity: Human, Mouse, Rat, Hamster, Monkey
Applications: Western blotting, Immunoprecipitation, Immunofluorescence, Immunohistochemistry, Flow Cytometer

Anti-Phospho-RIP1; https://www.cellsignal.jp/products/primary-antibodies/phospho-rip-ser166-antibody/31122
Species specificity: Mouse
Applications: Western blotting

Anti-RIP3; https://www.novusbio.com/products/ripk3-rip3-antibody_nbp1-77299#reviews-publications
Species specificity: Human, Mouse, Rat
Applications: Western Blot, Enzyme-linked immuno-sorbent assay, Immunocytochemistry, Immunofluorescence, Immunohistochemistry, Immunoprecipitation, Gel Supershift Assay

Anti-Phospho-RIP3; https://www.abcam.co.jp/products/primary-antibodies/rip3-phospho-s232-antibody-epr9516n-25-ab195117.html
Species specificity: Mouse
Applications: Enzyme-linked immuno-sorbent assay, Western Blotting, Dot blot

Immunohistochemistry:
Anti-CD45; https://www.biolegend.com/ja-jp/products/purified-anti-mouse-cd45-antibody-102?GroupID=BLG1932
Species specificity: Mouse
Applications: Flow Cytometer, Mass Cytometry, Immunohistochemistry, Immunoprecipitation, Western blotting, Complement Mediated Cell Depletion

Anti-CD68; https://www.bio-rad-antibodies.com/monoclonal/mouse-cd68-antibody-fa-11-mca1957.html?f=purified&JSESSIONID_STERLING=F3483C1D473E9F509EF33E1DC09EF821.ecommerce2&evCntryLang=JP-ja&cntry=JP&thirdPartyCookieEnabled=true
Species specificity: Mouse
Applications: Flow Cytometer, Immunohistochemistry, Immunoprecipitation, Immunofluorescence, Western blotting

Anti-Ly-6G/Ly-6C; https://www.bdbiosciences.com/ja-jp/products/reagents/microscopy-imaging-reagents/immunohistochemistry-reagents/purified-rat-anti-mouse-ly-6g-and-ly-6c.550291
Species specificity: Mouse
Applications: Flow Cytometer, Immunohistochemistry

Anti-CD3; https://www.abcam.co.jp/products/primary-antibodies/cd3-antibody-sp7-ab16669.html
Species specificity: Mouse, Rat, Human, Sheep, Rabbit, Horse, Chicken, Cow, Cat, Dog, Pig, Baboon, Woodchuck
Applications: Flow Cytometer, Immunohistochemistry, Western blotting

Anti-HMGB1; https://www.abcam.co.jp/products/primary-antibodies/hmgb1-antibody-ab18256.html
Species specificity: Mouse, Rat, Human, Rabbit, Cow
Applications: Immunofluorescence, Western blotting

Anti-α-sarcomeric actin; https://www.sigmaaldrich.com/JP/ja/product/sigma/a2172
Species specificity: Rat, Human, Sheep, Rabbit,Carp, Guinea Pig, Snake, Cow, Frog
Applications: Enzyme-linked immuno-sorbent assay, Immunohistochemistry, Western blotting

Animals and other research organisms

Policy information about studies involving animals; ARRIVE guidelines recommended for reporting animal research, and Sex and Gender in Research

Laboratory animals

Pla2g6-/-, +/+, Gpr34-/- mice were generated in our laboratory. C57BL/6JJmsSlc mice were purchased from SLC.
All mice were housed in a temperature and humidity-controlled (23 ± 1.5 °C, 45 ± 15%) room with 12-hr light-dark cycle (lights on from 8:00 to 20:00).
The sex and age for all the strains of mice were male and 10 to 14-week-old, respectively.
1-day-old Kwl/Wistar male rats were purchased from Kiwa Laboratory Animals.
All rats were used for neonatal rat cardiomyocyte isolation on the day of purchase.

Wild animals	The study did not involve wild animals.
Reporting on sex	Only male mice and rats were used in this study.
Field-collected samples	This study did not involve samples collected from the field.
Ethics oversight	All experimental protocols were approved by the Animal Research Committee of Osaka University. All in vivo and in vitro experimental protocols were carried out under the supervision of the Animal Research Committee at Osaka University and in accordance with the Guidelines for Animal Experiments at Osaka University and the Japanese Animal Protection and Management Law. These experiments were performed in accordance with the U.K. Animals (Scientific Procedures) Act 1986 and its associated guidelines, Directive 2010/63/EU for animal experiments. We have complied with ARRIVE (Animal Research: Reporting of In Vivo Experiments) guidelines.

Note that full information on the approval of the study protocol must also be provided in the manuscript.
